# Event and Comment

**Published:** 1915-07

**Authors:** 


### DENTAL REGISTER

Vol. LXIX JULY 1915 No. 7

### EVENT AND COMMENT

PANAMA-PACIFIC DENTAL CONGRESS. In this issue we print the final call and programme for the Congress of Dentists from all parts of the world at San Francisco, August 30th. That a strenuous effort has been made to prepare a meritorious programme of scientific papers and artistic exhibits can be clearly seen by reference to the programme published in another part of this journal. The dentists of the Pacific Coast will exert themselves to make the meeting memorable in every possible way, and we are sure that all who can go will be greatly benefited and pleased. For lavish hospitality the western dentists can't be excelled. Unquestionably the great war will prevent a large foreign attendance, but the favorable weather and excellent traveling facilities ought to assure a record-breaking attendance. The English Congress last year was practically a failure because of the war, but our congress should be a great success, even though it may be dependent on our own country for most of the programme. Most of our summer meetings have been concentrated on this one great gathering and it will be interesting to see what our country can produce when it is almost entirely thrown on its own resources.

UNIVERSITIES DECIDE ON CURRICULUM. A meeting of ten representatives of the great universities of this country having Dental Departments met in Chicago, June 24th and reaffirmed the action of the Dental Faculties Association of American Universities to begin a four years'

course of study for the degree of Doctor of Dental Surgery on or before the session beginning October, 1917. The meeting was attended by official representatives of all the universities and it passed a resolution that in the added year only a limited amount of academic or so called ancillary subjects should be allowed. On the assumption that there should be 1,200 working or class hours to be scheduled in a nine-months course or a total of 4,800 in the four-year curriculum, it was decided that not more than one-eighth, or 600 hours, should be devoted to academic subjects; such as rhetoric, physics, biology, foreign languages or mechanical drawing, etc. These subjects are not only most excellent cultural studies, but they lead up directly to the scientific subjects which are fundamentally the basis of all medical and dental science education. From the discussion provoked at the meeting it was evident that for the present, at least, a compromise course will have to be given to satisfy the traditional ideals developed from intensive devotion to the technical features of our art. We shall have two years in which to prepare for the inauguration of the four-year course, and it seems as though we should develop a more ideal course of instruction than we now have. There are many inconsistencies in our present curriculum that should not be carried over into the new one. It will doubtless be inexpedient to predict or to even seek uniformity in this matter, and perhaps because of the great differences in teaching faculties it might detract from ultimate results to require a uniform curriculum for all schools. We had hoped that the added year might have been one of purely academic or scientific studies, that the dental student might become a more conscientious student and thinker as well as a *doer*. The average high-school graduate is usually not a great thinker. His memory has been fairly well taught and disciplined, but adaptability, or initiative, does not seem to belong to him naturally or by training. A year or two of selected university work should transfer a high school boy to the dental school with a good memory, and a fair equipment of knowledge, and besides he should have learned to get at the principles involved in his art and have mental capacity to translate them into his own consciousness. It will take another decade perhaps to adjust ourselves to the four-year curriculum, and to be ready to make another forward step. It is to be hoped that we shall make few errors and that we may soon arrive at a proper conclusion.

